# Supplementary material for: MicroRNAs profiling in malaria and arbovirus coinfection: A systematic review protocol
Source: PLoS One. 2026 Jan 8;21(1):e0340672. doi: 10.1371/journal.pone.0340672 (PMC12782442; doi:10.1371/journal.pone.0340672)
Supplement: S2 File — Detailed search strategy for one major database (PubMed, Scopus, Web of Science, CENTRAL), which will be adapted for other databases. (DOCX) [file pone.0340672.s002.docx]

**MicroRNAs profiling in malaria and arbovirus coinfection: A systematic review protocol**

Search strategies query

| **Database** | **Query** |
| --- | --- |
| PubMed | ((((((((((((((malaria[Title/Abstract]) OR (Plasmodium falciparum[Title/Abstract])) AND (arboviral diseases[Title/Abstract])) OR (Dengue[Title/Abstract])) OR (Dengue fever[Title/Abstract])) OR (chikungunya[Title/Abstract])) OR (chikungunya fever[Title/Abstract])) OR (Zika[Title/Abstract])) OR (zika fever[Title/Abstract])) AND (microRNAs[Title/Abstract])) OR (microRNA[Title/Abstract])) OR (miRNAs[Title/Abstract])) OR (miRNA[Title/Abstract])) AND (miR[Title/Abstract])) OR (miRS[Title/Abstract]) |
| Scopus | ( TITLE-ABS-KEY ( micrornas ) AND TITLE-ABS-KEY ( malaria ) AND TITLE-ABS-KEY ( arbovirus ) OR TITLE-ABS-KEY ( arbovirus AND infections ) OR TITLE-ABS-KEY ( dengue ) OR TITLE-ABS-KEY ( dengue AND fever ) OR TITLE-ABS-KEY ( dengue AND virus ) OR TITLE-ABS-KEY ( chikungunya ) OR TITLE-ABS-KEY ( chikungunya AND fever ) OR TITLE-ABS-KEY ( chikungunya AND virus ) OR TITLE-ABS-KEY ( zika ) OR TITLE-ABS-KEY ( zika AND virus AND infection ) ) |
| Web of sciences | (((((((TS=(malaria)) AND TS=(microRNAs)) AND TS=("dengue infection")) OR TS=(dengue virus)) OR TS=(chikungunya)) OR TS=(chikungunya virus)) OR TS=(zika)) AND TS=(zika virus) and Review Article (Exclude – Document Types) and Letter or Proceeding Paper or Editorial Material (Exclude – Document Types) |
| CENTRAL | "malaria" in Title Abstract Keyword AND microRNAs in Title Abstract Keyword AND "dengue fever" in Title Abstract Keyword AND "chikungunya virus" in Title Abstract Keyword AND "Zika virus" |
