# Supplementary material for: MicroRNAs profiling in malaria and arbovirus coinfection: A systematic review protocol
Source: PLoS One. 2026 Jan 8;21(1):e0340672. doi: 10.1371/journal.pone.0340672 (PMC12782442; doi:10.1371/journal.pone.0340672)
Supplement: S3 File — Template for summarizing the assessment of the certainty of evidence using the GRADE approach. (DOCX) [file pone.0340672.s003.docx]

**MicroRNAs** **profiling in malaria and arbovirus coinfection: A systematic review protocol**

**S3 file**: Assessing the quality of evidence and the strength of recommendations.

| **Types of studies** | **Risks of bias** | **Interpretation** | **Quality of evidence** | **Strength of the recommendation** |
| --- | --- | --- | --- | --- |
| Randomized studies | Low risk of bias | Most information is from studies at low risk of bias. | High | Strong |
|  | Unclear risk of bias | Most information is from studies at low or unclear risk of bias. | Moderate | Moderate |
|  |  |  | Low |  |
| Non-randomized studies | High risk of bias | The proportion of information from studies at high risk of bias is sufficient to affect the interpretation of results. | Very Low | Weak |
