# Supplementary material for: MicroRNAs profiling in malaria and arbovirus coinfection: A systematic review protocol
Source: PLoS One. 2026 Jan 8;21(1):e0340672. doi: 10.1371/journal.pone.0340672 (PMC12782442; doi:10.1371/journal.pone.0340672)
Supplement: Attachment — Submitted filename: Response to the Reviewers.docx [file pone.0340672.s005.docx]

**Response to the Reviewers**

Manuscript ID: PONE-D-25-47997

MicroRNAs profiling in malaria and arbovirus coinfection: A systematic review protocol.

We thank the editors and reviewers for their thoughtful comments and constructive suggestions on our manuscript. We have carefully revised the manuscript to address all points raised. The changes are highlighted in the "Revised Manuscript with Track Changes" file. Below is our point-by-point response.

**Journal requirements revisions**

In addition to the specific points raised by the reviewers, we have also ensured full compliance with PLOS ONE's journal formatting requirements:

-The ethics statement has been placed exclusively in the 'Ethics and dissemination' subsection within the Methods.

-Formal captions for all Supporting Information files have been added at the end of the manuscript.

-The reference list has been thoroughly checked and formatted according to the Vancouver/ICMJE style.

**Academic Editor Comments**

**Comment:** *This manuscript describes a systematic review protocol that will characterize the profiles of microRNAs involved in malaria and arbovirus coinfections, which will advance understanding of coinfection pathogenesis. Besides miRNAs, siRNAs or dsRNAs also regulate gene expression, especially the first nanoparticle encapsulated dsRNA was reported in mosquitoes. Therefore, could nanoparticles also be employed to encapsulate miRNAs as well? Maybe the recent review (Recent progress in nanoparticle-mediated RNA interference in insects: Unveiling new frontiers in pest control) could be used to discuss this possibility.*

**Response:** We thank the editor for this insightful suggestion regarding the potential of nanoparticle-based delivery systems for miRNAs. While our systematic review protocol is primarily focused on the identification and profiling of miRNA signatures, we acknowledge the therapeutic potential of this technology. In response, we have integrated a mention of this promising future application into the abstract's conclusion to highlight the broader impact of our findings. ***Lines 47-48***

**Reviewer #1 Comments**

**Comment 1:** *I suggest this change in line 69-70: 'Pairing with the 3′-UTR appears to mediate post transcriptional silencing more effectively than pairing with the 5'-UTR or coding sequence, whereas miRNA interaction with promoter regions induces transcription [3].*

**Response:** We have rephrased the sentence as suggested for better clarity.

**Changes Made:** The sentence in the **Introduction** now reads: Pairing with the 3′-UTR appears to mediate post transcriptional silencing more effectively than pairing with the 5'-UTR or coding sequence, whereas miRNA interaction with promoter regions induces transcription [3]. ***Lines 74-76***

**Comment 2:** *In line 85 the author cite the viruses by their abbreviations (CHIKV, ZIKV, …) but you have never introduced them before. The first time you mention them, include the full name followed by the abbreviation in parentheses. Example: Chikungunya virus (CHIKV), Zika virus (ZIKV), Dengue virus (DENV). Thereafter, you can use just the abbreviation (for instance, “CHIKV replication…”).*

**Response:** We have now introduced the full virus names with their abbreviations upon first mention in the abstract.

**Changes Made:** Added in the **Abstract, Background section**: "Coinfections between malaria and arboviruses such as Dengue virus (DENV), chikungunya Virus (CHIKV), and Zika virus (ZIKV)..." ***Lines 23-24, 91-92***

**Comment 3:**  *In the search strategy/risk of bias assessment/data extraction and management paragraphs, I suggest replacing “we did” with an impersonal construction, which is more appropriate in an academic context. For example, instead of “We did an analysis,” write “An analysis was conducted.”*

**Response:** We have revised the methods section to use impersonal constructions throughout.

**Changes Made:** Changed to passive voice/impersonal constructions in the Search Strategy, Risk of Bias Assessment, and Data Extraction and Management sections. ***Lines 150-171; 184-224***

**Comment 4:** *"A conclusion paragraph is missing."*

**Response:** We have added a comprehensive Conclusion paragraph to the main text.

**Changes Made:** Added a new **Conclusion** section summarizing the protocol's objectives, methodology, and expected significance. ***Lines 325-335***

**Reviewer #2 Comments**

**Comment 1:** *"In addition to studies on co-infections, consider including studies investigating single infections (e.g., malaria or dengue) as a secondary analysis for meta-analysis. This may help identify disease-specific miRNAs and reduce heterogeneity in the overall synthesis."*

**Response:** We agree that including mono-infection studies will enhance the interpretation of co-infection-specific miRNA profiles. We have explicitly included this as a key part of our methodology.

**Changes Made:** Added a new subsection under **"Type of participants"** in the Inclusion Criteria detailing the inclusion of mono-infection studies for a secondary comparative analysis. ***Lines 120-125; 289-296***

**Comment 2:** *"Broaden the database searches and include preprint servers (e.g., medRxiv, bioRxiv) to capture recent or unpublished studies and minimize publication bias."*

**Response:** We have expanded our search strategy to include major preprint servers.

**Changes Made:** Added **medRxiv and bioRxiv** to the list of data sources in the **Search Strategy** and described the planned search process. ***Lines 154-155, 280***

**Comment 3:** *"Clearly describe how missing or ambiguous data will be addressed (e.g., by contacting study authors) to improve data reliability."*

**Response:** We have clarified the procedure for handling missing or unclear data.

**Changes Made:** Explicitly stated in the **"Statistical analyses and evidence synthesis"** section that corresponding authors will be contacted on a case-by-case basis to obtain clarifications. ***Lines 230-231***

**Comment 4:** *"Plan subgroup or sensitivity analyses based on infection type, study design, or detection methods to better interpret variability across studies."*

**Response:** We have now pre-specified plans for subgroup and sensitivity analyses to proactively investigate sources of heterogeneity.

**Changes Made:** Added a new dedicated paragraph in the **"Statistical analyses and evidence synthesis"** section detailing planned subgroup analyses by infection type, study design, detection method, sample type, and risk of bias. ***Lines 234-245***

We believe these revisions have significantly strengthened the manuscript and thank the editors and reviewers for their valuable input.
