# Supplementary material for: MicroRNAs profiling in malaria and arbovirus coinfection: A systematic review protocol
Source: PLoS One. 2026 Jan 8;21(1):e0340672. doi: 10.1371/journal.pone.0340672 (PMC12782442; doi:10.1371/journal.pone.0340672)
Supplement: Attachment — Submitted filename: Response to Reviewers.docx [file pone.0340672.s006.docx]

**Rebuttal letter: response to the Editor**

Manuscript ID: PONE-D-25-47997

MicroRNAs profiling in malaria and arbovirus coinfection: A systematic review protocol.

Dear Dr. Jisheng Liu,

Thank you for the opportunity to revise our manuscript and for the constructive feedback from the editorial team. We have carefully considered the comments and have made revisions to strengthen the manuscript accordingly. Please find below our point-by-point response.

**Response to Journal Requirements**

1. We have reviewed the reviewer comments and confirm that no specific citation recommendations were made that require the addition of new references.

2. We have thoroughly reviewed our reference list to ensure it is complete and correct. We confirm that our manuscript contains no retracted articles. As part of our revision to address the editor's comment, we have added four new references to the Discussion section.

**We have also corrected bibliographic information for reference 23, which was previously inaccurate, and updated the access dates for references 13, 16, and 22.**

**Response to the Academic Editor:**

**Comment:** In abstract L47-48, "Additionally, the miRNA profiles identified may provide foundations for developing novel therapeutic approaches, including nanoparticle-based delivery systems. This claim should be indicated in the discussion with supporting citations, as well as in the conclusion paragraph.”

**Response:** We thank the editor for this important suggestion. We agree that the therapeutic potential of the identified miRNA profiles requires proper contextualization within the existing literature. In response:

1. We have expanded the **Discussion section** in the paragraph now on *page 11 (lines 307-318)* to explicitly address the translational potential of miRNA signatures. We now discuss how dysregulated miRNAs can serve as therapeutic targets, citing relevant examples from other fields (Citation 24-25) and outline the rationale for nanoparticle-based delivery systems to overcome key challenges in RNA therapy, such as stability and targeted delivery (Citation 26-27).

2. We have confirmed and slightly reinforced this point in the **Conclusion** paragraph *(page 15, line 345-348)* to ensure it is presented as a clear and supported implication of our proposed systematic review.

These changes provide the necessary scholarly support for the claim made in the Abstract and enhance the manuscript's discussion of future applications.

We have also performed a final check to ensure the reference list is complete and correct.

We believe these revisions have significantly improved the manuscript and hope it now meets the high publication standards of PLOS ONE.

Thank you again for your time and consideration.

Sincerely,

Deutou Wondeu
