# Supplementary material for: MicroRNAs profiling in malaria and arbovirus coinfection: A systematic review protocol
Source: PLoS One. 2026 Jan 8;21(1):e0340672. doi: 10.1371/journal.pone.0340672 (PMC12782442; doi:10.1371/journal.pone.0340672)
Supplement: Attachment — Submitted filename: Response_to_Reviewers_auresp_3.docx [file pone.0340672.s007.docx]

**Response to Academic Editor**

**Comment:** The authors claim "Nanoparticle-based systems, particularly lipid nanoparticles have emerged as a leading solution". Some sentences regarding various nanomaterials with recent supported reference should be added before this sentence, including chitosan, star polycations, carbon quantum dots, layered double hydroxides guanylated polymers branched amphiphilic peptide capsules, cell-penetrating peptides etc. Then the author can narrow down to lipid-based nanoparticles. This will provide general information to specific for readers.

**Response:** We sincerely thank the Academic Editor, Dr. Liu, for this precise and valuable suggestion, which stems from his deep expertise in the field. We fully agree that providing this technological context will benefit readers.

In direct response, we have revised the relevant sentence in the **Discussion section**. We now explicitly state that a broad range of nanocarrier platforms-encompassing the very categories mentioned (natural polymers, inorganic materials, peptide-based systems, etc.) have been engineered for nucleic acid delivery, as **recently and comprehensively reviewed for RNA interference applications (new Reference 26 and 27)**. This provides the perfect foundational context to then logically focus on lipid nanoparticles as a leading therapeutic delivery platform.

We are confident that this addition, anchored by your authoritative review, fully addresses the comment and enhances the manuscript's discussion of future translational potential.
